# A fast look-up method for Bayesian mean-parameterised Conway–Maxwell–Poisson regression models

**DOI:** 10.1007/s11222-023-10244-0

**Published:** 2023-05-18

**Authors:** Pete Philipson, Alan Huang

**Affiliations:** 1 School of Mathematics, Statistics & Physics, Newcastle University, Newcastle upon Tyne, NE1 7RU UK; 2 School of Mathematics and Physics, University of Queensland, St Lucia, Queensland 4066 Australia

**Keywords:** Count data, Overdispersion, Underdispersion, Conway–Maxwell–Poisson, Numerical look-up table

## Abstract

Count data that are subject to both under and overdispersion at some hierarchical level cannot be readily accommodated by classic models such as Poisson or negative binomial regression models. The mean-parameterised Conway–Maxwell–Poisson distribution allows for both types of dispersion within the same model, but is doubly intractable with an embedded normalising constant. We propose a look-up method where pre-computing values of the rate parameter dramatically reduces computing times and renders the proposed model a practicable alternative when faced with such bidispersed data. The approach is demonstrated and verified using a simulation study and applied to three datasets: an underdispersed small dataset on takeover bids, a medium dataset on yellow cards issued by referees in the English Premier League prior to and during the Covid-19 pandemic, and a large Test match cricket bowling dataset, the latter two of which each exhibit over and underdispersion at the individual level.

## Introduction

Regression models for count data appear widely in the statistical (and other) literature, with classic treatise from Hilbe (2011) and Cameron and Trivedi (2013). Count data are commonly referred to as being overdispersed–relative to the Poisson distribution–when the variance exceeds the mean, and underdispersed when the mean exceeds the variance, with the former phenomenon much more widespread and typically handled via a negative binomial regression model, or other choices, such as the log normal or inverse Gaussian as mixing distributions for the underlying Poisson rate parameter.

For the less common, and thus less studied, situation of underdispersion, viable models are harder to come by and the most ostensibly promising candidate distributions have some clear drawbacks: they are hampered by a restricted parameter space (generalised Poisson), are not parameterised in such a way as to make standard inferences (gamma count and Conway–Maxwell–Poisson) or are difficult to evaluate (hyper-Poisson, Conway–Maxwell–Poisson and Poisson–Tweedie) due to the nature of the likelihood; this issue is naturally exacerbated in a Bayesian context.

Moreover, when dealing with hierarchical data, the two types of dispersion often occur together at some granular level (i.e. individuals), in what we refer to as bidispersion, within the same dataset. Under this scenario, a Poisson model is both conservative and anti-conservative for the cases of overdispersion and underdispersion respectively, whereas a negative binomial model will be anti-conservative for those individuals that are underdispersed - what is required is a model that can traverse the dispersion spectrum in either direction (within the same model), whilst still allowing for standard inferences and sufficiently small computational overhead so as to encourage routine use.

The Conway–Maxwell–Poisson (CMP) distribution allows for bidispersion, but contains an awkward normalising constant and is not parameterised in terms of its mean. The standard CMP model has probability mass function

1 $$\begin{aligned} \Pr (Y = y \mid \lambda , \nu ) = \frac{\lambda ^y}{(y!)^{\nu }} \frac{1}{G(\lambda , \nu )} \end{aligned}$$

where the normalising constant term $G(\lambda , \nu ) = \sum _{r=0}^{\infty } \lambda ^r/(r!)^{\nu }$ ensures that the CMP distribution is proper, but complicates analysis. The distribution is governed by a rate parameter, $\lambda \;(>0)$ and a dispersion term, $\nu (\ge 0)$, that allows for both over- ($\nu < 1$) and underdispersion ($\nu > 1$). Despite being introduced almost sixty years ago, motivated by a queueing problem (Conway and Maxwell 1962), the CMP distribution has been mostly overlooked in the statistical literature, although it has gained some popularity in the last twenty years or so with a broad range of interesting applications, from household consumer purchasing traits (Boatwright et al. 2003), retail sales, lengths of Hungarian words (Shmueli et al. 2005), road traffic accident data (Lord et al. 2008, 2010), life history, spatial and community ecology (Lynch et al. 2014), dispersion in positron emission tomography (Santarelli et al. 2016) and forecasting tropical cyclones (Mitchell and Camp 2021).

The wider applicability of the CMP distribution was demonstrated by Guikema and Goffelt (2008) and Sellers and Shmueli (2010), who illustrated that the CMP distribution can be incorporated into the generalised linear modelling framework for both Bayesian and frequentist settings respectively, and through the development of an R package (Sellers et al. 2019) in the case of the latter, to help facilitate routine use. Alternative methods to circumvent intractability for the standard CMP model in the Bayesian setting have been developed by Chanialidis et al. (2018), who used rejection sampling based on a piecewise enveloping distribution and more recently (Benson and Friel 2020), who developed a faster method using a single, simple envelope distribution, but these approaches do not routinely generalise to the mean-parameterised CMP distribution introduced below.

The orthodox parameterisation in terms of a rate, $\lambda$, that does not correspond to the mean, has restricted the wider usage of the CMP regression model and has led to the development of a mean-parameterised Conway–Maxwell–Poisson (MPCMP) distribution (Huang 2017) whereby

2 $$\begin{aligned} \mu = \sum _{r=0}^{\infty } \frac{r \lambda ^r}{(r!)^{\nu }G(\lambda , \nu )}, \end{aligned}$$

where $\mu$ denotes the mean. This parameterisation comes at a computational cost, however, since implementation of the model now requires the solution of a polynomial equation for $\lambda$ of the form

3 $$\begin{aligned} \sum _{r=0}^{\infty } (r - \mu ) \frac{\lambda ^r}{(r!)^{\nu }}= 0. \end{aligned}$$

The probability mass function for the MPCMP distribution replaces $\lambda$ in (1) with the solution of (3)

4 $$\begin{aligned} \Pr (Y = y \mid \lambda (\mu , \nu ), \nu ) = \frac{\lambda (\mu , \nu )^y}{(y!)^{\nu }} \frac{1}{G\left\{ \lambda (\mu , \nu ), \nu \right\} } \ , \end{aligned}$$

where $\lambda (\mu , \nu )$ reflects the dependence of the rate parameter on both $\mu$ and $\nu$ when solving the polynomial in (3). Adopting this parameterisation brings all of the flexibility and interpretation of a standard generalised linear model, including the orthogonality of the mean and dispersion parameters (Huang and Rathouz 2017) and allows intuitive regression models for both the mean and dispersion components and the incorporation of offsets if required, placing it on a similar inferential footing as classic count models.

A hybrid bisection and Newton–Raphson approach to find $\lambda$ was proposed by Huang (2017), implemented in R package mpcmp (Fung et al. 2019)–buttressed by its subsequent inclusion in the flexible glmmTMB package (Brooks et al. 2017) - and applied in small sample Bayesian settings (Huang and Kim 2019), whereas Ribeiro et al. (2018) suggested an asymptotic approximation of $G(\lambda , \nu )$ to obtain a closed form estimate for $\lambda$, again replete with an accompanying R package (Elias Ribeiro Junior 2021). The appeal of the former is its more exact nature, but this can incur considerable computational costs in large sample settings since the iterative approach would be required at each MCMC iteration, and, potentially, for a large number of (conditional) mean values.

The approximation used by Ribeiro et al. (2018) is conceptually appealing due to its simplicity and computational efficiency, but is likely to be inaccurate for some of the combinations of $\mu , \nu$ encountered in general, and the level of accuracy will also vary across these combinations. In particular, the approximation is less accurate for $\nu > 1$, for which counts are underdispersed and the Conway–Maxwell–Poisson distribution may be most useful, and when $\lambda < 10^{\nu }$ (Bonat et al. 2017), which is likely to occur regularly in real data settings with small counts; indeed, it is the case in each application considered here. Irrespective of approach, the infinite sum also needs to be approximated by a finite sum–we return to this point in Sect. 3.1.

## Bayesian MPCMP models

We consider a general MPCMP model for an observed data sample $y_1 \ldots , y_n$ of size *n*, where we adopt a log-linear model for both the mean and dispersion parameters. Namely, we have

$$\begin{aligned} \log \mu _i = \varvec{x}_{1i}^T \varvec{\beta } \end{aligned}$$

and

$$\begin{aligned} \log \nu _i = \varvec{x}_{2i}^T \varvec{\gamma } \end{aligned}$$

where $\varvec{\beta }$ and $\varvec{\gamma }$ are vectors of regression parameters of length *p* and *q* with associated covariate vectors $x_{1i}$ and $x_{2i}$ for the mean and dispersion components respectively and $i = 1, \ldots , n$ in each case. Clearly, the models for the mean and dispersion may, and are indeed likely, to have covariates in common, but there is no requirement for this to be so. Under general independent prior distributions $\pi (\varvec{\beta })$ and $\pi (\varvec{\gamma })$ for the regression parameters we can formulate the posterior distribution as

5 $$\begin{aligned} \pi (\varvec{\beta }, \varvec{\gamma } \mid \varvec{y})&= \pi (\varvec{\beta })\pi (\varvec{\gamma }) \prod _{i=1}^n f(y_i \mid \varvec{\beta }, \varvec{\gamma }) \\ {}&= \pi (\varvec{\beta })\pi (\varvec{\gamma })\nonumber \times \prod _{i =1}^n \frac{\lambda _i\left( e^{\varvec{x}_{1i}^T \varvec{\beta }}, e^{\varvec{x}_{2i}^T \varvec{\gamma }} \right) ^{y_i}}{(y!)^{e^{\varvec{x}_{2i}^T \varvec{\gamma }}} G\left\{ \lambda _i\left( e^{\varvec{x}_{1i}^T \varvec{\beta }}, e^{\varvec{x}_{2i}^T \varvec{\gamma }}\right) , e^{\varvec{x}_{2i}^T \varvec{\gamma }}\right\} } \end{aligned}$$

Focusing on the mean parameters we have

6 $$\begin{aligned}&\pi (\varvec{\beta }\mid \varvec{\gamma }, \varvec{y}) \propto \pi (\varvec{\beta }) \nonumber \times \prod _{i =1}^n \frac{\lambda _i\left( e^{\varvec{x}_{1i}^T \varvec{\beta }}, e^{\varvec{x}_{2i}^T \varvec{\gamma }} \right) ^{y_i}}{(y!)^{e^{\varvec{x}_{2i}^T v{\gamma }}} G\left\{ \lambda _i\left( e^{\varvec{x}_{1i}^T \varvec{\beta }}, e^{\varvec{x}_{2i}^T \varvec{\gamma }}\right) , e^{\varvec{x}_{2i}^T \varvec{\gamma }}\right\} } \nonumber \\ \end{aligned}$$

and we then work on the log-scale to circumvent possible numerical issues when evaluating the above to obtain

7 $$\begin{aligned} \log \left\{ \pi (\varvec{\beta }\mid \varvec{\gamma }, \varvec{y})\right\} \propto \log \left\{ \pi (\varvec{\beta })\right\}&+ \sum _{i=1}^n y_i \log \left\{ \lambda _i\left( e^{\varvec{x}_{1i}^T v{\beta }}, e^{\varvec{x}_{2i}^T \varvec{\gamma }}\right) \right\} \\ \nonumber&- \log \left[ G\left\{ \lambda _i\left( e^{\varvec{x}_{1i}^T \varvec{\beta }}, e^{\varvec{x}_{2i}^T \varvec{\gamma }}\right) , e^{\varvec{x}_{2i}^T \varvec{\gamma }}\right\} \right] . \end{aligned}$$

In an analogous fashion, the log-posterior distribution of the dispersion parameter vector $\varvec{\gamma }$ is given by

8 $$\begin{aligned} \log \left\{ \pi (\varvec{\gamma }\mid \varvec{\beta }, \varvec{y})\right\} \propto \log \left\{ \pi (\varvec{\gamma })\right\} \nonumber&+ \sum _{i=1}^n y_i \log \left\{ \lambda _i\left( e^{\varvec{x}_{1i}^T \varvec{\beta }}, e^{\varvec{x}_{2i}^T \varvec{\gamma }}\right) \right\} \nonumber \\ {}&- \log \left[ G\left\{ \lambda _i\left( e^{\varvec{x}_{1i}^T \varvec{\beta }}, e^{\varvec{x}_{2i}^T \varvec{\gamma }}\right) , e^{\varvec{x}_{2i}^T \varvec{\gamma }}\right\} \right] \nonumber \\ {}&- e^{\varvec{x}_{2i}^T \varvec{\gamma }} \log (x!), \end{aligned}$$

where additional dependence on the dispersion parameters is captured through the final term.

From Eqs. (7) and (8) we can see that, in each case, evaluation of the log-posterior depends on $\lambda _i$, which itself is a function of the mean, $\mu _i$ and dispersion, $\nu _i$ in both the second (directly) and third (since the normalising constant itself is also a function of $\lambda _i$) terms. We note that the posterior distribution for the dispersion parameters depends additionally on the (inexpensive to evaluate) log-factorial term involving the data, which is not required in Poisson and negative binomial models. In a model without covariates (in either component) we would only require a scalar $\lambda$, but this is clearly unrealistic in any wider regression setting. As such, we typically need to evaluate $\lambda _i$ multiple times at each iteration, incurring considerable computational costs.

## Alternative approaches for MPCMP models

Since $\lambda$ is positive, there is a single sign change in (3) when $\mu > r$, which, by Descartes’ rule of signs, informs us that there is a solitary positive real root. Hence, a solution for $\lambda$ can be found without recourse to approximations or less scaleable iterative methods and we can directly solve the $k^{th}$ order polynomial using the R function polyroot, for some suitably chosen *k*, which makes use of the Jenkins-Traub algorithm.

This may in itself offer computational gains over previous methods if incorporated as part of an iterative scheme, but here we further propose to pre-compute a look-up table of $\lambda$ values (found using polyroot) which may offer more substantive reductions in computing time by front-loading some of the computational cost. Hence, the aim of the proposed approaches are to render the MPCMP model a practical alternative for count data that we would suggest as the *de facto* approach when faced with bidispersed data. As such, the two proposed methods are compared to the existing method established in the literature (and implemented in software packages) via a trio of simulation studies and associated applications.

### Computing the look-up table

We observe from Eq. (3) that the polynomial coefficients for the $\lambda$ terms depend on the parameters $\mu$ and $\nu$. Considering the mean $\mu$ is itself modelled via a log-link and that we are typically interested in computing the log-likelihood rather than the likelihood, we can generate sequences for both $\log \mu$ and $\nu$ to form a two-dimensional look-up table and solve the polynomial in $\log \lambda$ for each unique choice of $(\log \mu , \nu$).

Clearly, a coarse table may compromise accuracy, whereas a finer table will take longer to compute. We find that, in practice, a step size of 0.01 for $\mu$ ranging between 0 and 32 (more than thrice the largest observable count across the simulations and applications) and $\nu$ ranging from 0 to 10, is sufficient to ensure accuracy for our applications, in conjunction with interpolation within the look-up table and extrapolation outside the table–see Sect. 3.2.

Such a table takes around twenty seconds to compute (using R version 4.2.2 R Core Team 2022 on a 2022 MacBook Pro), consisting of over 850*K* items. A further advantage of this approach is that the calculations can be done in parallel by making use of the foreach (Microsoft and Weston 2020) and doParallel (Corporation and Weston 2019) packages across multiple cores–this gives considerable gains in this case and such an approach would be even more beneficial for a larger, or finer, table, or both. The code to generate the look-up table is provided on https://github.com/petephilipson/MPCMP_grid/blob/main/grid.R. The look-up table would be supplied as a simple text file to users, something that will be made use of in development of an R package.

The time to compute the look-up table is minimal in this instance and is easily absorbed into the overall computing time. If a finer table was found to be required then the time taken will necessarily scale up; we find it takes around five minutes for a step size of 0.001, where parallelisation is of greater value.

However, this is a one-off procedure; realistically most models are run several times while analysts add or remove parameters, tune their MCMC models by monitoring and tweaking acceptance rates and assessing convergence diagnostics amongst other things. As such, the total time taken for analysis *en bloc*, even for a small dataset, is unlikely to be substantially inflated even if a finer look-up table is required. Indeed, for large enough datasets the computing cost of generating the look-up table will be absorbed even on a one-off analysis when compared to existing methods.

In this work we truncated the infinite series in Eq. (3) at around twice the maximum value of $\mu$, $\mu _{MAX}$, used in constructing the look-up table, i.e. $k \approx 60$ and found this to be suitable for the simulations and applications under consideration; results were reassuringly robust to a choice of $k \approx 90$. This is likely due to the low counts (and moderate underdispersion) found in each of our real world settings, upon which the generated synthetic data are based.

We note that when the counts have a truncated upper limit (as in the third application) then we simply take $k = y_{\tau }$ where $y_{\tau }$ denotes this truncation value. This, in turn, induces a finite limit on the normalising constant and circumvents concerns around defining the upper bound for the look-up table on $\mu$.

### Interpolation and extrapolation

To improve numerical accuracy of the look-up method, we employ bilinear interpolation for values within the look-up table. Explicitly, for any given input ($\mu ,\nu$) the corresponding $\log \lambda$ value is approximated by

$$\begin{aligned}{} & {} \log \lambda (\mu , \nu ) \approx \log \tilde{\lambda }(\mu , \nu ) \\{} & {} \quad =\begin{bmatrix} (1 - \Delta _\mu ) \\ \Delta _\mu \end{bmatrix}^\top \begin{bmatrix} \log \lambda (\mu _1, \nu _1) &{} \log \lambda (\mu _1, \nu _2) \\ \log \lambda (\mu _2, \nu _1) &{} \log \lambda (\mu _2, \nu _2) \end{bmatrix} \begin{bmatrix} (1 - \Delta _\nu ) \\ \Delta _\nu \end{bmatrix} \, \end{aligned}$$

where $\mu _1,\mu _2$ and $\nu _1, \nu _2$ are the floor and ceiling tabulated values of the mean and dispersion surrounding the given input $(\mu , \nu )$, respectively, and

$$\begin{aligned} \Delta _\mu = \frac{\log \mu - \log \mu _1}{\log \mu _2 - \log \mu _1} \ , \qquad \Delta _\nu = \frac{\nu - \nu _1}{\nu _2 - \nu _1} \end{aligned}$$

are the corresponding weights.

For input values that lie outside the pre-computed table, we utilise bilinear *extrapolation* to obtain an approximation for $\lambda$ in an analogous way. We note that such extrapolation was not required in the simulations and applications that follow, though it acts as a safeguard for general deployment of this approach. The fact that we do not need to extrapolate here suggests that the look-up table boundaries are sensible.

An alternative approach to using a look-up table with interpolation and extrapolation is via an associative array, or hash table, where the $\mu$ and $\nu$ values would form a key-pair that can be looked up to fetch the corresponding value of $\lambda$. The advantage of an associative array (or hash table) is that there are no pre-specified limits (unlike for a two-dimensional matrix that represents our look-up table) and keys that are not in the current table can be added ‘on-the-fly’. The R package hashmap (Russell 2017) provides such functionality and was considered here. However, the time taken to perform the look-up was considerably larger than using the proposed (fixed) look-up table of values augmented with inter(extra)polation as necessary so this approach is not considered further here.

## Simulation studies

The mean-parameterised Conway–Maxwell–Poisson distribution is not included (nor, for that matter, is the orthodox CMP parameterisation) in mainstream ‘off-the-shelf’ Bayesian software such as rstan (Stan Development Team 2020) and jags (Plummer 2004) so bespoke code was written in R to implement the models. The code is available on GitHub at https://github.com/petephilipson/MPCMP_grid/tree/main/Fitting. All simulations and applications were carried out in R 4.2.2 (R Core Team 2022) on a 2022 MacBook Pro with 16GB RAM.

We consider performance of the proposed look-up table method, the method of solving the polynomial within the MCMC scheme and the current de facto bisection method under three simulation scenarios, with each foreshadowing applications to three real-world datasets in Sect. 5; we refer to the three approaches as ‘bisection’, ‘polynomial’ and ‘look-up’ in the results that follow. The scenarios consider what we regard as small ($n = 150$), moderate ($n = 2000$) and large ($n = 15000$) sample sizes. The latter two scenarios also consider individual level effects on the mean and dispersion owing to the nature of the applications upon which they are based; for the small sample we consider scalar $\nu$ only.

In each simulation study we conduct $N = 100$ replicates, each with $K=5000$ MCMC iterations, and make use of the R package mpcmp (Fung et al. 2019) to generate the simulated counts in each case. We use the simulation scenarios to demonstrate both the accuracy and speed of the proposed method, the latter of which is shown via a comparison to the previously used approach by Huang and Kim (2019); to ensure fair comparisons, the code used is identical in all regards apart from the evaluation of $\lambda$. The code to generate the simulation scenarios is available at https://github.com/petephilipson/MPCMP_grid/blob/main/sim_studies.R.

### Simulation I

The first simulation scenario considers a small sample size of $n=150$ and is based on the takeover bids data which is subsequently analysed in Sect. 5.1. In particular we consider $Y_i \sim \text {MPCMP}(\mu _i, \nu )$ where we have a log-linear model for the mean rate

$$\begin{aligned} \log (\mu _i) = \varvec{x}_{1i}^T \varvec{\beta }. \end{aligned}$$

Here, $\varvec{\beta }$ is a parameter vector of length ten, comprising of an intercept term and nine regression parameters. The covariate vector $\varvec{x}_{1i}$ is made up of five binary covariates (generated as Bernoulli random variables with $p = 0.5$ in each case) and four further continuous covariates, one from the normal distribution and two from the lognormal distribution, one of which is also included in its squared form. The (scalar) dispersion parameter is set as $\nu = 1.5$, indicating moderate underdispersion.

**Fig. 1:**
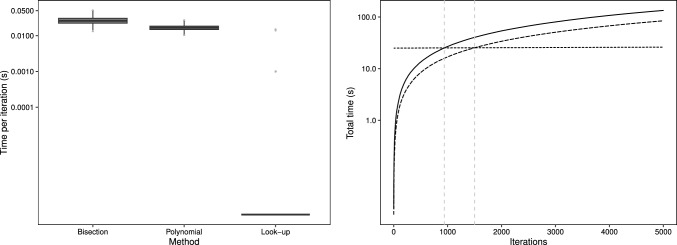
Average time (in seconds) per iteration of the MCMC scheme for the three methods for simulation I (left panel); cumulative time (in seconds) to perform 5K iterations for each method (right panel) with the solid line for bisection, long-dashed line for polynomial and short-dashed line for look-up. The vertical dashed lines represent the iteration number at which the total time for the look-up method is first less than the other methods

The time taken per iteration is considerably lower for the look-up approach, as exhibited in the left panel of Fig. 1 where we have used the log-scale for better visual comparisons; results in terms of parameter estimation are included in the Appendix–see Fig. 10. The mean (SD) time per iteration is 0.027 (0.009), 0.017 (0.004) and 0.0002 (0.0006) for the bisection, polynomial and look-up methods respectively, with the proposed look-up table approach being some 135 and 85 times faster in this setting at the iteration level than the bisection and polynomial methods respectively, which are themselves broadly similar.

The right panel of Fig. 1 displays the cumulative run time against iterations, where we have incorporated the pre-calculation of the look-up table into the time to ensure comparisons are fair. Upon completion of 940 iterations the look-up method starts to outperform the bisection method, and after around 1500 iterations the look-up method becomes the fastest of the three methods. Hence, even in a small sample setting if a modest run of only 2000 iterations is desired then the look-up method is the fastest.

However, notwithstanding these gains, when considering the total time taken to perform 5000 iterations, we observe that, whilst the look-up table method is appreciably quicker, the bisection and polynomial approaches are not overly time-consuming in this small sample scenario, with total times of around 134 (bisection) and 85 (polynomial) seconds, compared to the look-up table runtime of 24 s. To this end, we now consider two further settings with larger sample sizes in order to investigate how the proposed method performs in larger data settings.

### Simulation II

In the second simulation we consider a sample size of $n = 2000$ based on the data analysed in Sect. 5.2. In this scenario the counts come from $m = 20$ individuals, each with one hundred observations; note that this balanced design is an artefact of the simulation and not a requirement. Hence, we now consider (repeated) count responses $Y_{ij} \sim \text {MPCMP}(\mu _{ij}, \nu _i)$ where we have further introduced an individual specific heterogeneity parameter $\nu _i$ to recognise that the dispersion–along with the mean rate–may vary between individuals. We adopt a log-linear model for the mean rate for individual *i* at response *j* of the form

$$\begin{aligned} \log (\mu _{ij}) = \beta _0 + \beta _1 x_{1ij} + \beta _2 x_{2ij} + \beta _3 x_{1ij} x_{2ij} + \theta _i \end{aligned}$$

where both $x_{1ij}$ and $x_{2ij}$ are binary covariates with associated parameters $\beta _1$ and $\beta _2$, with their interaction further captured by $\beta _3$. The individual effects on the mean rate and dispersion are captured through $\theta _i$ and $\nu _i$ respectively, with $i = 1, \ldots , 20$ in each case. In the simulation the true parameter values are given by $\varvec{\beta } = (1.00, -0.10, -0.20, 0.10)$ with $\varvec{\theta }$, generated using a sequence, as $(-0.475, -0.425, \ldots , 0.425, 0.475)$ with magnitudes loosely based on the second application. Finally, although the dispersions can vary by individual, we simulate using two values for $\nu _i$, one representing overdispersion ($\nu _i = 0.8, i = 1, \ldots , 10$) and the other underdispersion ($\nu = 1.25, i =11, \ldots , 20$) for simplicity.

**Fig. 2:**
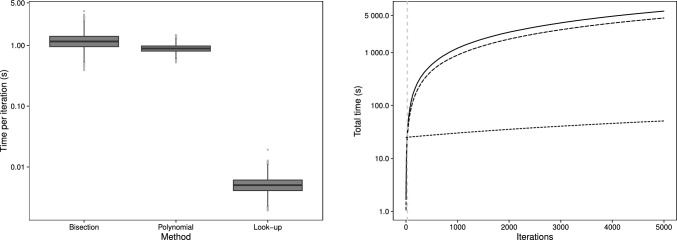
Average time (in seconds) per iteration of the MCMC scheme for the three methods for simulation II (left panel); cumulative time (in seconds) to perform 5K iterations for each method (right panel) with the solid line for bisection, long-dashed line for polynomial and short-dashed line for look-up. The vertical dashed lines represent the iteration number at which the total time for the look-up method is first less than the other methods

Figure 2 displays boxplots of the time taken (on the log-scale for clarity) per iteration across the 100 simulations. We see that times are once again considerably higher using the bisection and polynomial approaches than for the look-up table method. The mean (SD) time per iteration is 1.206 (0.285), 0.897 (0.121) and 0.005 (0.005) for the three respective methods, with the look-up table approach now exhibiting a 250-fold decrease in CPU time per iteration (or a relative difference of 99.6%) over the bisection method, and a near 180-fold decrease with the polynomial method, based on the average iteration time.

The right panel of Fig. 2 displays the cumulative run time against iterations, where, as before, we have absorbed the pre-calculation of the look-up table into the total time for the look-up method. Upon completion of around only 30 iterations the look-up method starts to outperform the other methods.

Boxplots of the posterior distrbutions for each of the fixed effects, and for the individual effects on both the mean and dispersion are presented in Figs. 11 and  12. In each case we see that the method performs well in terms of estimating the true parameter values.

### Simulation III

The third simulation is for a larger sample size of $n = 15000$, broadly based on the data analysed in Sect. 5.3. Here the counts come from $m = 100$ individuals, each with 150 observations, whereby $Y_{ij} \sim \text {MPCMP}(\mu _{ij}, \nu _i)$ in what amounts to a direct extension of the second simulation, albeit with an additional covariate. We adopt a log-linear model for

$$\begin{aligned} \log (\mu _{ij}) = \beta _0 + \beta _1 x_{1ij} + \beta _2 x_{2ij} + \beta _3 x_{3ij} + \beta _4 x_{4ij} + \theta _i \end{aligned}$$

where each $x_{mij}, m = 1, \ldots , 4$ is a binary covariate with associated parameters $\beta _m$. The individual effects on the mean rate and dispersion are captured through $\theta _i$ and $\nu _i$ respectively, with $i = 1, \ldots , 100$ in each case. In the simulation the true parameter values are given by $\varvec{\beta } = (1, -0.10, 0.05, 0.10, 0.15)$ with $\varvec{\theta }$, generated using a sequence from $-0.475$ to 0.475 with magnitudes loosely based on the third application. Finally, we simulate the dispersions using a sequence of values for $\nu _i$, ranging from overdispersion ($\nu _i = 0.8$) through to underdispersion ($\nu = 1.25$).

**Fig. 3:**
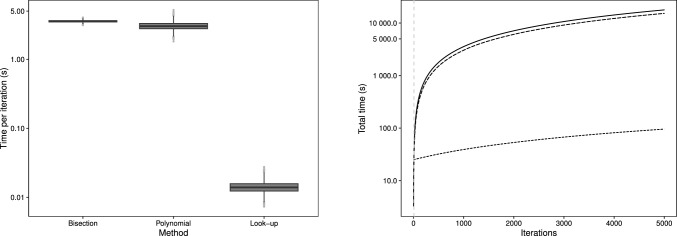
Average time (in seconds) per iteration of the MCMC scheme for the three methods for simulation III (left panel); cumulative time (in seconds) to perform 5K iterations for each method (right panel) with the solid line for bisection, long-dashed line for polynomial and short-dashed line for look-up. The vertical dashed lines represent the iteration number at which the total time for the look-up method is first less than the other methods

The time in seconds per iteration (on the log-scale) for each method under simulation III are displayed in the left panel of Fig. 3. We see that times are once again lowest for the look-up table approach, followed by the polynomial and bisection methods; the mean (SD) time per iteration is 3.56 (0.12), 3.08 (0.81) and 0.014 (0.006) for the three respective methods. The look-up table approach now results in a more than 99% decrease in CPU time per iteration compared to both alternative methods.

The right panel of Fig. 3 displays the cumulative run time against iterations, where, as before, we have absorbed the pre-calculation of the look-up table into the total time for the look-up method. After only 8 and 9 iterations the look-up method starts to outperform the bisection and polynomial methods respectively, suggesting the look-up method offers better gains in terms of reduced computational times as the sample size increases. Boxplots of the posterior distrbutions for the covariates, $\varvec{\beta }$ and individual level means and dispersions are given in Figs. 13 and 14.

### Comparison of computing times for simulations

We now compare the computing times per iteration across the sample sizes governing the simulations to allow an insight into the scaleability of the proposed look-up method vis a vis the other methods under consideration.

**Fig. 4:**
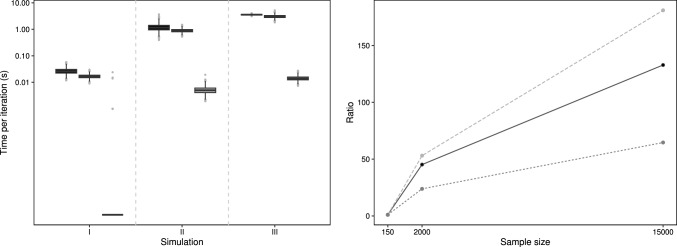
Average time (in seconds) per iteration of the MCMC scheme for each method (bisection–dark grey, polynomial–medium grey, look-up–light grey) across the three simulation settings (left panel); times are on the log-scale for clarity. Ratio of mean time taken for an iteration when compared to simulation I (right panel) for each method (bisection–solid, polynomial–long dash, look-up–short dash)

The right-hand panel of Fig. 4 allows an assessment of the scaleability with sample size for each approach, where each method is relatively compared in terms of mean time taken to perform an iteration with the smallest sample size setting of simulation I.

The look-up approach takes 23% longer as we move from $n=150$ to $n=2000$, with a further three-fold increase when we consider $n = 15,000$. For the bisection method, the mean time taken for an iteration is around forty times longer when $n=2000$ compared to $n=150$, with a further three-fold increase as we move to $n=150,00$. For the polynomial method, there is near fifty-fold increase in the average iteration time as we go from $n = 150$ to $n = 2000$ and an approximate three-fold increase for the largest sample size. We note here that this serves only as a guide since the postulated model changes across the simulations; in particular, we consider only a scalar disperson in simulation I.

Across the three simulations we can clearly see from the left panel of Fig. 4 that the look-up table approach offers considerable computational gains, to the extent that the mean time per iteration is lower for the look-up method with a sample size of $n = 150,00$ than using the other methods with the smallest sample size of $n = 150$.

**Table 1:** Approximate effective sample sizes for the look-up method across the three simulations

Simulation	Comparison method	$m_e$ (SD)
I	Bisection	998 (0.31)
	Polynomial	999 (0.20)
II	Bisection	995 (1.86)
	Polynomial	995 (1.86)
III	Bisection	999 (2.43)
	Polynomial	999 (2.21)

### Effective sample sizes for look-up method in simulations

In order to quantify the error induced by the proposed method, we consider the approximate effective sample size when compared to both the bisection method and solving the polynomial directly. Based on Eq. (5) we can find the approximate posterior, based on the approximate value of $\lambda$ from the interpolated look-up table, as:

$$\begin{aligned} \tilde{\pi _{}}(\varvec{\beta }, \varvec{\gamma } \mid \varvec{y}) \propto \pi (\varvec{\beta })\pi (\varvec{\gamma }) \prod _{i=1}^n\frac{\tilde{\lambda _i} (\mu _i, \nu _i)^{y_i}}{y_i!^{\nu _i} G(\tilde{\lambda _i} (\mu _i, \nu _i), \nu _i)} \end{aligned}$$

where $\tilde{\lambda _{}}$ is the interpolated value from the look-up table. We can then form the ratio

9 $$\begin{aligned} r(\varvec{\beta }, \varvec{\gamma }) :=\frac{\pi (\varvec{\beta }, \varvec{\gamma } \mid \varvec{y})}{\tilde{\pi _{}}(\varvec{\beta }, \varvec{\gamma } \mid \varvec{y})} \end{aligned}$$

where the numerator is given in Eq. (5). Here we consider both the bisection method and the polynomial as the numerator in Eq. 9. We then define the approximate effective sample size (Owen 2013) as

$$\begin{aligned} m_e = \frac{\left( \sum _{i=1}^K r(\varvec{\beta }, \varvec{\gamma })\right) ^2}{\sum _{i=1}^K r(\varvec{\beta }, \varvec{\gamma })^2} \end{aligned}$$

where *K* is the number of MCMC samples under consideration.

We calculate the approximate effective sample size under each simulation scenario based on $N = 100$ replicates (this allows us to quantify the uncertainty in the estimate). In each case we consider the approximate effective sample size based on a reference value of $K = 1000$, with results–rounded to the nearest integer - presented in Table 1. We see that the look-up table gives approximate effective sample sizes that are very close to the nominal $K=1000$ value, indicating that the interpolated $\lambda$ values are very close to their more exact counterparts found under the bisection and polynomial methods.We note that this procedure, whilst having some overlap with the standard ESS calculation, is a comparison between the look-up table and both the bisection and polynomial approaches and does not imply that the ESS values would be this high, as measured in the usual way (i.e. as the number of independent realisations from the target distribution based on a sample of 1000 values).

## Applications

The proposed method is illustrated on three data analysis examples, a small sample exhibiting underdispersion, and both medium and large datasets, which each exhibit underdispersion and overdispersion due to individual level heterogeneity–the latter two datasets are available at https://github.com/petephilipson/MPCMP_grid. In each case we first obtain the MLE of the model parameters using the mpcmp package (Fung et al. 2019) to provide starting values and, moreover, to find the estimated covariance matrix for some of the model parameters that require block updates. For analysis, four MCMC chains were run in parallel with 1000 warm-up iterations followed by 5000 further iterations.

### Takeover bids data

In this first application we revisit the underdispersed takeover bids data first presented in Cameron and Johansson (1997) and subsequently analysed via a hyper-Poisson model (Sáez-Castillo and Conde-Sánchez 2013) and an MPCMP model in both a frequentist and Bayesian setting (Huang 2017; Huang and Kim 2019). In particular we focus on the latter analysis and compare the proposed look-up approach with this previous analysis–full details of the dataset are given in both of the former two references and the data are publicly available from the R packages Ecdat (Croissant and Graves 2020) and mpcmp (Fung et al. 2019).

The regression model for the log mean rate is formulated as

$$\begin{aligned} \log (\mu _i) = \beta _0 + \beta _1 x_{i1} + \ldots + \beta _9 x_{i9} \end{aligned}$$

for $i = 1, \ldots 126$. Some background details on the explanatory variables $\varvec{x_1}, \ldots , \varvec{x_9}$ are given in the supplementary materials. The dispersion parameter, $\nu$, is considered to be a scalar for these data to match previous analyses, but covariates could be incorporated in practice.

The vector of parameters $\varvec{\beta } = (\beta _1, \ldots , \beta _9)$ is jointly updated with a proposal covariance matrix based on the estimate obtained using the mpcmp package. For consistency, the (vague) priors are the same as those used in Huang and Kim (2019):

$$\begin{aligned} \varvec{\beta }\sim & {} N(0, 10^{5}I_9) \\ \nu\sim & {} LN(0, 10^{5}). \end{aligned}$$

#### Results

**Fig. 5:**
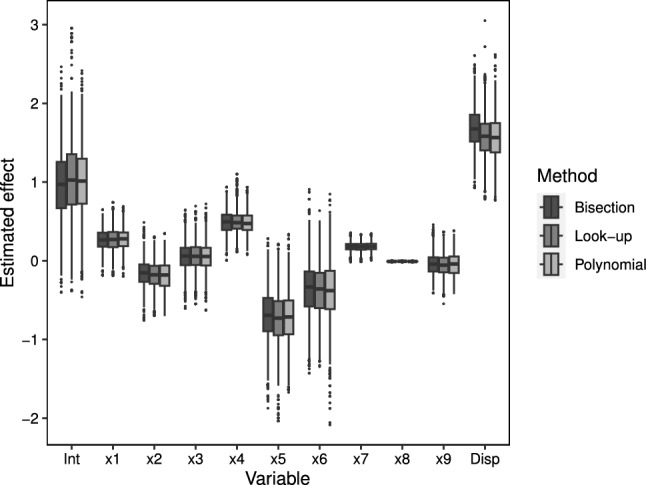
Posterior distributions of estimated parameters (on the log-scale) for the takeover bids data for the bisection and look-up methods

Boxplots of the posterior parameter estimates for the takeover bids data are given in Fig. 5 under each approach. Results are near identical across the three approaches and are in keeping with those presented in Huang and Kim (2019). These results can be interpreted multiplicatively on the mean rate–as in classic count data models–by exponentiating, but we do not go in to detail here. Broadly, though, firms who were open to friendly third-party bids had a higher average count of bids, whereas firms with a lower price and holding less stock had a lower average count of bids. For other parameters, the highest posterior density interval covered zero.

We also monitor the CPU time taken to perform a single iteration of the MCMC scheme under each approach, allowing us to investigate the mean and standard deviation. The mean (SD) times (in seconds) to complete a single iteration were 0.0002 (0.0007) and 0.017 (0.007) for the look-up and bisection methods respectively, with the look-up method being almost 100 times faster on average–see the conclusion of Sect. 5. The polynomial method performed very similarly to the bisection method in this case, albeit with a smaller standard deviation.

### Yellow cards shown by English Premier League match referees

We consider data on the number of yellow cards shown by referees in the men’s English Premier League between seasons 2018/19 and 2020/21. This gives a dataset of 1140 matches and we have two observations for each match (one each for the home and away team) to give a dataset of size $n = 2280$ comprising of $m = 25$ referees. The original data are publicly available at https://www.football-data.co.uk/. We consider individual level effects for both components of the model alongside the effect of being at home and on whether the absence of fans due to the COVID-19 pandemic made a material difference to the number of cards shown, and whether this was different for nominal home and away teams in matches played. Hence we model the log mean rate for the number of yellow cards given by referee *i* in their $j^{th}$ match to the home ($k=1$) or away ($k=2$) team as

$$\begin{aligned} \log (\mu _{ijk})&= \beta _0 + \beta _1 I(\text {Home}_{ijk} = 1) + \beta _2 I(\text {NoFans}_{ijk} = 1) \\&+ \beta _3 I(\text {Home}_{ijk} = 1) \times I(\text {NoFans}_{ijk} = 1) + \theta _i \end{aligned}$$

where $\varvec{\beta }$ is the vector of parameters for the intercept, the effect of home advantage, the effect of no fans and the interaction between home advantage and there being no fans present. The individual referee effects (on the mean) are captured through $\theta _i, i = 1, \ldots 25$. We consider dispersion at the individual (referee) level in this application through $\nu _i, i = 1, \ldots , 25$.

**Fig. 6:**
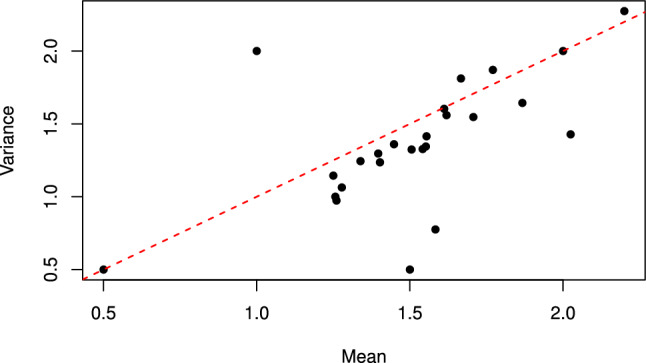
Observed variance against observed mean for each referee data; the dashed red line represents equidispersion

As way of motivation, we plot the observed variance against the observed mean for each referee in Fig. 6. Points below the line represent referees for whom the raw data are underdispersed, with points above showing overdispersion. We observe that many of the referees exhibit underdispersion, but that some may also be overdispersed, motivating use of a model that can handle both types.

The vectors of mean parameters $\varvec{\beta } = (\beta _0, \beta _1, \beta _2, \beta _3)$ and individual effects $\varvec{\theta } = (\theta _1, \ldots , \theta _{25})$ are each jointly updated with proposal covariance matrices based on the estimates obtained using the mpcmp package. The priors are chosen to reflect that large differences–considered to be a doubling or halving of the rate at which cards are issued–in referees and in home and behind closed doors conditions are thought to be unlikely, with a $5\%$ chance of being outside this range. This leads to the choices

$$\begin{aligned} \varvec{\beta } \sim N(\varvec{0}, 0.5\log 2 \times I_9) \end{aligned}$$

and

$$\begin{aligned} \varvec{\theta } \sim N(\varvec{0}, 0.5\log 2 \times I_{25}) \end{aligned}$$

The heterogeneity parameters have component-wise updates, via a normal random walk on the log-scale through the introduction of $\alpha _i = \log (\nu _i)$. We adopt a prior with a mean of equidispersion (on the log-scale), namely $\alpha _i \sim N(0, 0.25)$ for $i = 1, \ldots , 25$ for the heterogeneity parameters.

#### Results

We find evidence that fewer yellow cards were administered on average behind closed doors with a posterior mean for $\beta _2$ of $-$0.12 ($-$0.22, $-$0.03), suggesting that matches are played with less physical intensity, or that there is an overall crowd effect. Furthermore, whilst more yellow cards are traditionally given to the away team with a posterior mean for $\beta _1$ of $-$0.07 ($-$0.13, $-$0.01), likely owing to crowd pressure on the referee, where this is ameliorated in the absence of fans, with the posterior mean for $\beta _3$ of 0.04 ($-$0.06, 0.14).

**Fig. 7:**
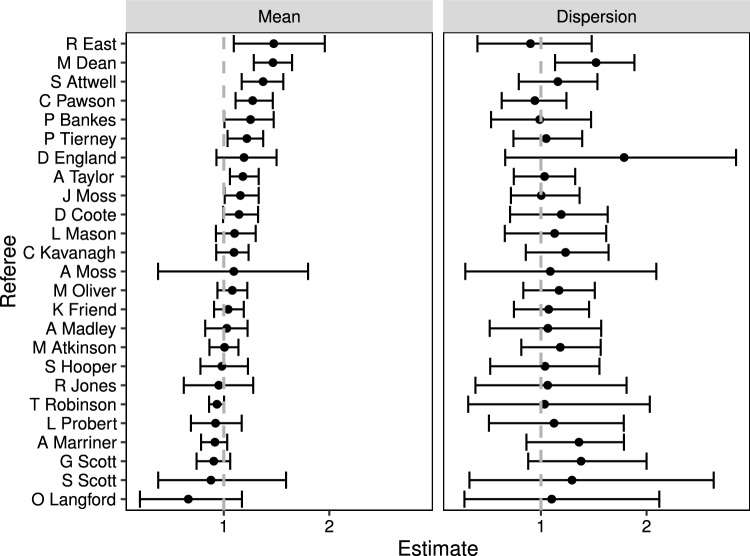
Posterior HDIs for individual referee effects (left panel) and for individual referee dispersions (right panel) in descending mean order; the dashed, vertical line indicates the average rate or equidispersion accordingly

At the referee level, we see from Fig. 7 that a handful of referees issue more yellow cards on average, led by Mike Dean with a posterior mean of 1.38 (1.22–1.52) and one referee, Andre Marriner, issues significantly fewer yellow cards on average with a posterior mean of 0.88 (0.76–0.99). In terms of dispersion, we see that the majority of referees exhibit underdispersion when looking at their posterior mean values (which mostly lie above one), although the levels of uncertainty are quite broad. Interestingly, Mike Dean also has the largest posterior mean for undersdispersion of 1.41 (1.05–1.77). A comparison of computing times for this application is given in Fig. 9 at the end of the section.

### Test match cricket bowling data

In this third application we consider Test match cricket bowling data. In particular we focus on the $n = 115$ players who have taken more than 150 wickets in their Test career; the data are available from http://www.howstat.com/cricket/home.asp. We define $Y_{ij}$ to represent the number of wickets taken by player *i* in during his *j*th bowling performance, and let $n_i$ denote the number of observations in the career of player *i*. Using this notation, we have the model $Y_{ij} \sim \text {MPCMP}(\mu _{ij}, \nu _{i})$ with $i=1,\ldots , 115$ and $j=1,\ldots ,n_i$.

The other main aspect of the data to note is that they are aggregated counts for each bowler. Alongside the wickets there are data available on the venue (home or away, coded as 0/1 respectively), the (within) match innings (coded ordinally as 1–4) and on the number of runs conceded for each count of wickets taken. These are all considered as covariates in the model. We also have data on the identity of the player, so allow for individual heterogeneity. This gives the model

$$\begin{aligned} \log \mu _{ij}&=\beta _0 + \beta _1 I(\text {Home}_{ij} = 1) \\&\quad + \beta _2 I(\text {Innings}_{ij} = 2) + \beta _3 I(\text {Innings}_{ij} = 3) \\&\quad + \beta _4 I(\text {Innings}_{ij} = 4) + \beta _5 \text {Runs}_{ij} + \theta _i \end{aligned}$$

where $\varvec{\beta }$ is the vector of parameters for the intercept, the effect of playing away from home and the effects of bowling in match innings two, three and four (hence bowling at home and in the first innings are set as the reference groups in this model) and for the continuous covariate runs.; some context for the covariates is given in the supplementary materials. The individual player effects (on the mean) are captured through $\theta _i, i = 1, \ldots 115$. Here, recognising that we may have both under and overdispersion at the individual level, we opt for a player-specific dispersion term $\nu _i, i = 1, \ldots 115$. Naturally, this could be extended to include covariates, particularly runs, but this facet of the model is not pursued further here.

We note in passing that the counts are truncated at ten in this application since there are a maximum of ten wickets to take in a given match innings, thereby circumventing any concerns about the range of $\mu$ in defining the look-up table and about the point at which to truncate the now finite series, which is truncated at ten.

A Metropolis-within-Gibbs algorithm is used in the MCMC scheme, with component-wise updates for all parameters except for those related to the game-specific effects, $\varvec{\beta }$. For these parameters a block update was used to circumvent the poor mixing seen when deploying one-at-a-time updates. A sum-to-zero constraint was used for the player ability effects, $\varvec{\theta }$. The priors are

$$\begin{aligned} \varvec{\beta }\sim & {} N(0, 0.5\log 2 I_6) \\ \varvec{\theta }\sim & {} N(\varvec{0}, 0.5\log 2 I_{115}) \\ \varvec{\nu }\sim & {} N(\varvec{0}, 0.5\log 3 I_{115}). \end{aligned}$$

#### Results

In the traditional cricket bowling average the number of runs conceded per wicket is reported, i.e. runs/wickets. since this places the metric on the same scale as the batting average. Statistically, however, it makes more sense to consider the reciprocal of this, with wickets as a count. Formulating this in a log-linear model would neccesitate the inclusion of the $\log (\text {Runs})$ terms as an offset. As such, we can assess the validity of the traditional average by inspecting the posterior distribution of $\beta _5$ which has mean (HDI interval) 0.41 (0.38–0.43)–this is a long way away from one, indicating that a proper treatment of the bowling average might consider a nonlinear relationship–in this case the expected count of wickets would be proportional to runs raised to the power of 0.41; we note that we have used a sample of Test bowlers so this result may not hold across all players.

The posterior mean for $e^{\beta _1}$ is 0.90 (0.88–0.92) suggests that there is a definite home advantage effect, to the extent that bowlers take $10\%$ fewer wickets on average playing away from home. The posterior distribution for the second and third innings effects ($\beta _2$ and $\beta _3$ respectively) have 95% intervals that include one, indicating there is no change to the rate at which wickets are taken in these innings. For the fourth innings, however, the posterior mean (for $e^{\beta _4}$) is 0.88 (0.84–0.92), which is counter to conventional wisdom about bowling being easier as the pitch gets older. However, these estimates are adjusted for the nonlinear form of runs mentioned above, under which (relatively) more wickets are expected to be taken for lower values of runs that are more common in the fourth innings.

**Fig. 8:**
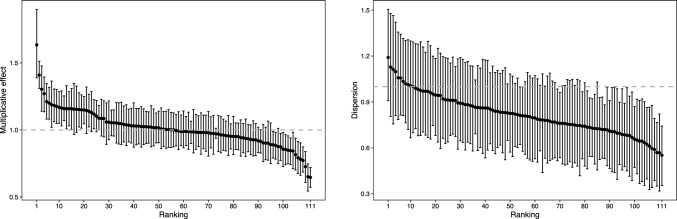
Ordered posterior HDIs for individual bowler means $\varvec{\theta }$ (left) and dispersions $\varvec{\nu }$ (right) in the Test match bowling application

Plots of the individual bowlers posterior highest density intervals for both their mean ability and dispersion parameters are given in Fig. 8. We find that around $10\%$ of players have a posterior mean value for $\nu _i$ that is consistent with underdispersion, reflecting that a model capable of handling dispersion in either direction is required here in order to accurately model these data. The top ten bowlers (ranked by posterior mean values of $\theta$) are presented in Table 2.

**Table 2:** Top ten bowlers from application III, ranked by posterior mean values of $\theta _i$

Rank	Player (country)	$E(\theta , \vert \, \nu ,y)$	$E(\nu \, \vert \, \beta ,\theta ,y)$
1	SF Barnes (England)	1.63 (0.13)	0.67 (0.15)
2	M Muralitharan (Sri Lanka)	1.41 (0.05)	0.86 (0.09)
3	CV Grimmett (Australia)	1.30 (0.09)	0.85 (0.15)
4	Sir RJ Hadlee (New Zealand)	1.27 (0.07)	0.66 (0.10)
5	MD Marshall (West Indies)	1.21 (0.06)	0.85 (0.12)
6	AA Donald (South Africa)	1.20 (0.06)	1.06 (0.13)
7	Yasir Shah (Pakistan)	1.19 (0.09)	0.68 (0.14)
8	DK Lillee (Australia)	1.18 (0.06)	0.91 (0.12)
9	R Ashwin (India)	1.18 (0.07)	0.78 (0.12)
10	DW Steyn (South Africa)	1.17 (0.06)	0.81 (0.11)

**Table 3:** Mean time in seconds required to perform a single iteration of the respective MCMC schemes for each method across the three applications

Application	Method	Mean time (SD) per iter in seconds
Takeover bids	Bisection	0.017 (0.007)
	Polynomial	0.017 (0.004)
	Look-up	0.0003 (0.0005)
Yellow cards	Bisection	0.932 (0.046)
	Polynomial	0.813 (0.016)
	Look-up	0.003 (0.002)
Test bowlers	Bisection	4.105 (1.401)
	Polynomial	4.866 (0.046)
	Look-up	0.010 (0.006)

### Comparison of computing times for applications

**Fig. 9:**
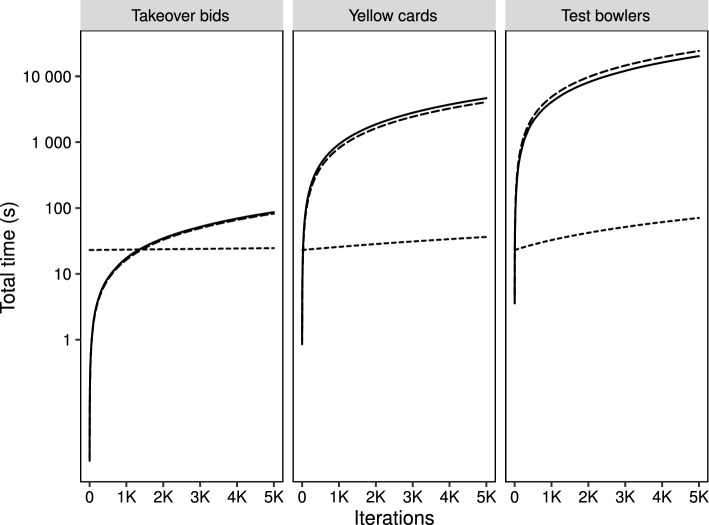
Cumulative time (in seconds, on the log-scale) taken to perform 5K iterations for each method and application. The solid line represents the bisection method, the long-dashed line is for the polynomial with the short-dashed line for the look-up approach

**Fig. 10:**
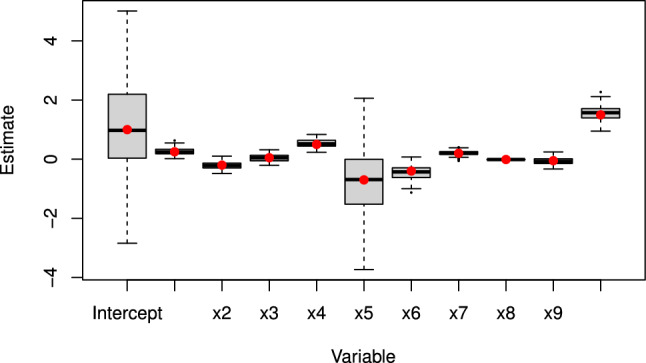
Boxplots of posterior distributions under simulation I for the look-up table approach–the red dots indicate the true values

**Fig. 11:**
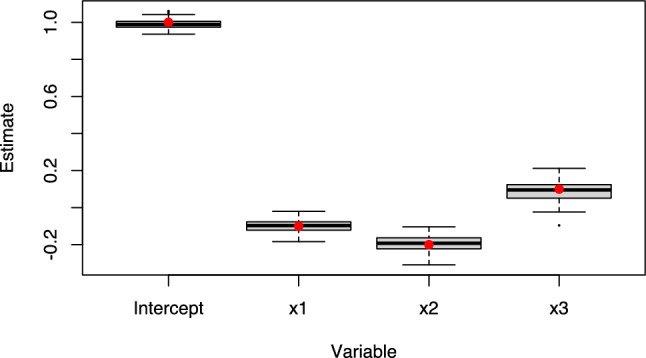
Boxplots of posterior distributions for $\varvec{\beta }$ under simulation II for the look-up table approach; the red dots indicate the true values

**Fig. 12:**
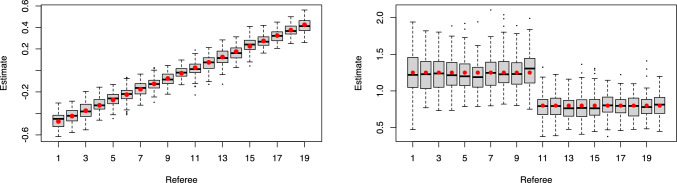
Boxplots of posterior distributions for $\varvec{\theta }$ (left) and $\varvec{\nu }$ (right) under simulation II for the look-up table approach; the red dots indicate the true values

**Fig. 13:**
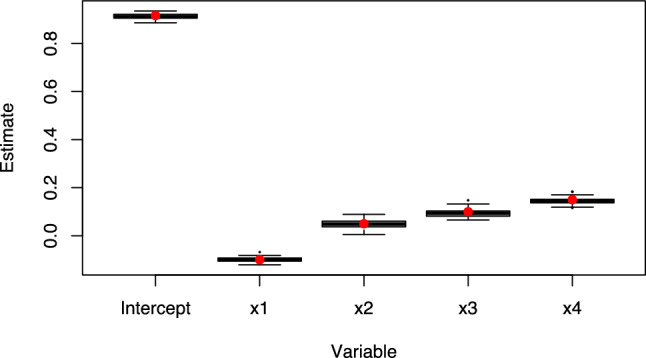
Boxplots of posterior distributions for $\varvec{\beta }$ under simulation III for the look-up table approach; the red dots indicate the true values

**Fig. 14:**
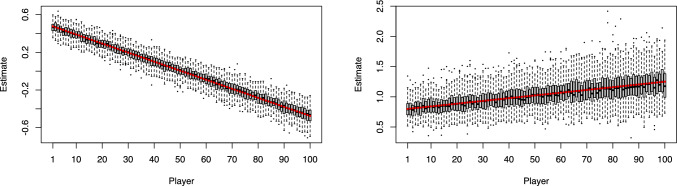
Boxplots of posterior distributions for $\varvec{\theta }$ (left) and $\varvec{\nu }$ under simulation III for the look-up table approach; the red dashed line indicates the true values

Comparing the computing times per iteration across the applications allows an insight into the scaleability of the proposed look-up method in real settings. The results echo the earlier findings from the simulation study, namely computing times are substantially lower when using the look-up approach (see Fig. 9) with mean (SD) times per iteration given across the applications in Table 3

In turn, these confer total fitting times in seconds–unless stated otherwise–based on 5000 iterations, after taking the pre-computation of the look-up table into account of: 87.35 (bisection), 82.57 (polynomial) and 24.53 (look-up) for the bids application; 4661.14 (bisection; 77.69 min), 4064.11 (polynomial; 67.74 min) and 36.64 s (look-up) for the yellow cards application and 20526.06 (bisection; 342.10 min), 24330.88 (polynomial; 405.52 min) and 71.56 (look-up).

This serves to emphasise that the look-up table method is fast even in large data settings, whereas the other methods do not scale well. For small datasets, the gains in speed, while appreciable, would not benefit the practitioner to a great extent but as we move to a moderate sample size, and reiterating that, oftentimes, models will be run multiple times, we see a meaningful difference of an order of magnitude as we move from tens of seconds to tens of minutes and this is exacerbated in the larger sample setting where we the look-up approach produces results in little more than a minute whilst the other approaches each take several hours.

We also calculated the approximate effective sample size under the look-up table method compared to both the bisection and polynomial methods in each application, in the same manner as for the simulations–see Sect. 4.5. For a notional sample size of $N=1000$, we found value of $m_e$ of 998, 996 and 996 for the takeover bids, yellow cards and bowling applications; results were the same when using either the bisection or polynomial approach as the comparison.

## Discussion

In this work we presented a fast look-up table Bayesian implementation of an MPCMP model capable of handling both under and overdispersion. The proposed model may have broader use in other fields where there may be bidispersed data, for example modelling goals scored in football matches–stronger and weaker teams are likely to exhibit underdispersion; hockey, ice-hockey and baseball all have small counts as outcomes of interest with bidispersion likely at the team or player level, or both.

Moving away from sports to other fields, the MPCMP model could be used to model parity, which is known to vary widely across countries with heavy underdispersion in more developed countries (Barakat 2016) and for longitudinal counts with volatile (overdispersed) and stable (underdispersed) profiles at the patient level, where the level of variability may be related to an outcome of interest in a joint modelling setting. Spatial models with small counts may also benefit from deployment of an MPCMP model (Li and Dey 2021) in place of the commonly adopted Poisson mixed with a lognormal distribution.

Classic count models are not capable of handling underdispersed data. However, there are alternative count models capable of handling both under- and overdispersion which may perform equally well, such as those based on the Poisson–Tweedie (Bonat et al. 2018), gamma count (Zeviani et al. 2014) and generalised Poisson (Consul and Famoye 1992) distributions; these models all have some restrictions (in estimation, interpretation and a restricted parameter space respectively) in handling underdispersion and were not pursued further here, but would benefit from future research.

The approach presented in this work may prove useful for other count data models (and beyond) where routine implementation is hampered by a normalising constant or where the mean is not a closed form function of the rate parameter such as the hyper-Poisson model of Sáez-Castillo and Conde-Sánchez (2013) and extensions (Chakraborty and Imoto 2016) and generalisations (Imoto 2014) of the Conway–Maxwell–Poisson distribution.

One drawback of the MPCMP model is that it is limited to geometric overdispersion, which proved more than sufficient for the range of applications considered here but may be a restriction in other settings. As such, future research to identify and implement an appropriate count data model capable of handling arbitrarily large over and underdispersion, whilst preserving standard inference and retaining computational feasibility, would offer complete flexibility for regression modelling of count data.

Avenues for future work are in the development of robust heuristics or theoretical bounds for the choices of *k* and upper limit of $\mu$ to pre-specify the number of terms involved in approximating the underlying polynomial and in the specification of the dimensions of the look-up table for the general case. The range of likely values for $\mu$ can be informed by the location of the count responses as is done here in an ad hoc manner, but for the dispersion it may be informative to reparameterise using the variance, which may also be preferable for inferential reasons.

## Appendix A: Further details on datasets used in the application section

Here we give further information and background for the covariates used in the takeover bids and Test match cricket bowling applications in Sect. 5

### Background on takeover bids data

The following descriptions of the explanatory variables are taken from Sáez-Castillo and Conde-Sánchez (2013) and Huang and Kim (2019):

- Defensive actions taken by management of target firm: indicator variable for legal defense by lawsuit ($x_1$), proposed changes in asset structure ($x_2$), proposed change in ownership structure ($x_3$) and management invitation for friendly third-party bid ($x_4$).
- Firm-specific characteristics: bid price divided by price 14 working days before bid ($x_5$), percentage of stock held by institutions ($x_6$), total book value of assets in billions of dollars ($x_7$) and book value squared ($x_8$).
- Intervention by federal regulators: an indicator variable for Department of Justice intervention ($x_9$).

### Background on test match cricket bowling data

The covariates included in the third application are game-specific, allowing for home advantage and pitch degradation (via the match innings effects). Home advantage is ubiquitous in sport (Pollard and Pollard 2005), and it is widely believed that it is easier to bowl as a match progresses to the third and fourth innings due to pitch degradation.

## Results from simulation studies

Boxplots of posterior distributions for each simulation are included here.

### Simulation I

We see from Fig. 10 that the proposed look-up table approach performs very well in terms of estimating the regression parameters and the heterogeneity parameter, even in this small sample setting.
